# Hospital crowdedness evaluation and in-hospital resource allocation based on image recognition technology

**DOI:** 10.1038/s41598-022-24221-6

**Published:** 2023-01-06

**Authors:** Lijia Deng, Fan Cheng, Xiang Gao, Wenya Yu, Jianwei Shi, Liang Zhou, Lulu Zhang, Meina Li, Zhaoxin Wang, Yu-Dong Zhang, Yipeng Lv

**Affiliations:** 1 School of Computing and Mathematical Sciences, The University of Leicester, University Road, Leicester, LE1 7RH UK; 2 School of Public Health, School of Medicine, Shanghai Jiao Tong University, Shanghai, People’s Republic of China; 3 Department of Endodontics, School and Hospital of Stomatology, Shanghai Engineering Research Center of Tooth Restoration and Regeneration, Tongji University, Shanghai, People’s Republic of China; 4 Department of Health Service, College of Health Service, Naval Medical University of the Chinese People’s Liberation Army, Shanghai, People’s Republic of China; 5 The First Affiliated Hospital, Hainan Medical University, Haikou, People’s Republic of China; 6 School of Management, Hainan medical university, Haikou, People’s Republic of China

**Keywords:** Health policy, Computer science, Information technology

## Abstract

How to allocate the existing medical resources reasonably, alleviate hospital congestion and improve the patient experience are problems faced by all hospitals. At present, the combination of artificial intelligence and the medical field is mainly in the field of disease diagnosis, but lacks successful application in medical management. We distinguish each area of the emergency department by the division of medical links. In the spatial dimension, in this study, the waitlist number in real-time is got by processing videos using image recognition via a convolutional neural network. The congestion rate based on psychology and architecture is defined for measuring crowdedness. In the time dimension, diagnosis time and time-consuming after diagnosis are calculated from visit records. Factors related to congestion are analyzed. A total of 4717 visit records from the emergency department and 1130 videos from five areas are collected in the study. Of these, the waiting list of the pediatric waiting area is the largest, including 10,436 (person-time) people, and its average congestion rate is 2.75, which is the highest in all areas. The utilization rate of pharmacy is low, with an average of only 3.8 people using it at the one time. Its average congestion rate is only 0.16, and there is obvious space waste. It has been found that the length of diagnosis time and the length of time after diagnosis are related to age, the number of diagnoses and disease type. The most common disease type comes from respiratory problems, accounting for 54.3%. This emergency department has congestion and waste of medical resources. People can use artificial intelligence to investigate the congestion in hospitals effectively. Using artificial intelligence methods and traditional statistics methods can lead to better research on healthcare resource allocation issues in hospitals.

## Introduction

Medical resources are limited, and the demand for medical resources continues to increase^1^. Various countries have varying degrees of shortage of medical resources, which is more severe in developing countries^2–4^. Rational allocation of macro-level health resources involves regional health planning, the layout of health resources, and so on. And the micro-level involves the rational use of inventory resources, which in medical institutions includes such things as the distribution of human resources, the setting of departments, and the layout of buildings. The direct manifestation of the distribution of medical resources in a hospital is the congestion situation in the hospital^5^. In recent years, the number of daily visits in urban hospitals, especially large general hospitals, has continued to increase^1^. And various public health emergencies in cities have occurred from time to time, resulting in an increasing frequency of congestion in hospitals^6^. This not only seriously affects the quality of medical services and reduces the satisfaction of patients, but also increases the possibility of medical disputes and aggravates the conflict between doctors and patients^7^. Therefore, in order to reduce and alleviate the congestion of the hospital and improve the patient experience, the hospital is trying to reform and optimize the allocation of hospital resources^8^.

Crowding occurs when the medical services required by patients exceed the capacity that the hospital can provide. Generally, Hospital design, department layout and resource allocation affect the congestion in the hospital^9^. Investigating the congestion in the hospital and studying the specific relationship between congestion and hospital design and system planning could provide managers with effective decision-making opinions on the allocation of medical resources.

Artificial intelligence is the study of how to make computers do intelligent tasks that only humans could do. In recent years, artificial intelligence has been widely used in the medical field as a new technology that plays an important role in medical practice such as imaging assisted diagnosis and pharmaceutical exploitation^10,11^. However, there is a lack of high-tech applications related to artificial intelligence in health management. At the same time, artificial intelligence has played a huge role in the field of public management and public security. For example, monitoring the crowd gathering through surveillance video to prevent stampedes and other gathering accidents^12^.

Similar to the need to monitor crowd aggregation and prevent congestion in the field of public safety, hospital management also needs to manage congestion. In this field, the management of congestion and allocation of resources in the existing hospitals usually relies on the personal experience of the administrators, without quantitative data analysis. Manual collection of crowd data for quantitative study in hospitals requires a lot of manpower and time. When dealing with resource allocation problems, AI can have better adaptability and responsiveness than manual scheduling^13,14^. Therefore, similar to use AI to monitor crowd aggregation and prevent congestion in the field of public safety, the use of advanced computer technology to manage hospital congestion is a feasible method.

The emergency department (ED), which has a diagnosis and treatment room of general clinics, internal medicine, surgery, gynecology, pediatrics, and other specialized departments, is a relatively independent unit. And the emergency department often requires receiving a mass patient, easily forming a short peak of visits^15^. Therefore, the emergency department is a perfect research object for congestion research. In order to provide an early alert for the manager and a reference for solving the congestion in the hospital, this research aims to use artificial intelligence technology to intelligently monitor and analyze the time and location characteristics of the hospital congestion, the relationship among different medical treatment processes and its’ influencing factors.

## Methods

### Study design and research object

This research is based on the existing hospital’s treatment process and big data of the crowd to analyze the congestion of the hospital and the rationality of their resource allocation. This research focus on the spatial and temporal distribution of the density of the crowd through artificial intelligence identification, collection and analysis. We collect data from a tertiary hospital. Tertiary care institution settings are adopted in China, and tertiary hospitals being the largest hospitals with the highest technology levels. Tertiary hospitals are similarly the most congested providers while having the most complete treatment link. Studying the congestion of tertiary hospitals is beneficial to study the congestion law of each diagnosis and treatment link in medical institutions. We randomly selected a tertiary hospital. We communicate with the emergency department of this hospital and conduct an on-site inspection, so as to understand the emergency procedures in the actual operation and the areas with large people flow. And we randomly selected a week of monitoring video records from the emergency department with 168 h of video, from 26 October to 1 November 2020, including the registered hall, the waiting area of Pediatrics, the waiting area of Pharmacy, the waiting area of the Internal medicine and Surgery, the waiting area of Inspection department. In addition, we get disease diagnosis-related information, including patient’s age, gender, disease diagnosis information and visit times. All information extracted does not involve basic information, such as individual names.

### Data collection

In this study, we create a model of patient flow in the hospital to distinguish the various areas of the hospital. The research divides the whole process of medical treatment into three parts: Registration, Visits and Examinations, and the Post-diagnosis process. Registration is divided into online registration and on-site registration. Only the patient’s arrival in the hospital is considered in the study. In this part, we collect the data from the registration hall of the hospital. The diagnosis part includes diagnostic rooms of all departments. Usually, the doctor arranges an examination for the patient after the initial consultation. And then, the patient returns to the clinic for further diagnosis after the examination. In this study, we focus on the time of initial diagnosis. Therefore, we collect the data from waiting areas of various departments in the hospital. The post-diagnosis process refers to the process after diagnosis until leaving the hospital. This part includes inspection, payment, taking medicine, etc. Based on this model, we collect three categories of information: (1) Image information: monitoring information at various nodes; (2) medical visit information, i.e. the visitor records of this emergency department; (3) Architectural information, i.e. the architectural situation in this emergency department.

Therefore, we focus on the actual scene of the emergency department and collect surveillance videos of crowded areas in the emergency department. The acquisition area to be collected includes emergency registration, the waiting area of the department of Pediatrics (the Department of Pediatrics in the emergency department is in an independent area), the department of internal medicine and Surgery, the inspection waiting area and an independent pharmacy. For each area, we collect the surveillance video of that area in one week in batches. The daily collection time of each area is the working time of the department. Of these, Pediatrics and internal medicine and Surgery are 24-h jobs, and registration offices, inspection departments, and pharmacies are from 8:00 a.m. to 6:00 p.m. Every half hour, we intercept a one-minute surveillance video. Therefore, the Pediatrics department and the department of internal medicine and Surgery collect 48 videos per day, while the other areas collect 21 videos per day. We collect 1113 videos in total.

The videos are divided according to the acquisition area, and the videos of different areas are saved in chronological order. Because all the areas we collect are waiting areas. Most people in the video are stationary. In order to reduce the total amount of data and accelerate the data processing, we choose a relatively low sampling rate. The area of each scene is large, the shortest length of the corridor is about 8 m. We assume that the walking speed through pedestrians is 1.5 m/s, so the sampling rate of 0.2 FPS may not miss the people who are passing through. Video frame extraction is performed at Frames Per Second (FPS) = 0.2, i.e., 1 image is collected every 5 s. 10 images are collected for each video. The images are divided according to the acquisition area and chronologically deposited into different folders. Finally, we get 11,130 images for crowd counting. The data collection at this stage provides a realistic basis for the next project design.

On the other hand, we also collate the records of emergency visits. This record contains the number of patients, gender, age, outpatient diagnosis, the earliest time of receipt, the earliest time of diagnosis, the earliest time of payment, and the earliest time of dispensing medication. This record can be combined with video data to provide more detail on studying the congestion in this emergency department.

### Image process based on convolutional neural network

We use a convolutional neural network (CNN) to measure the number of people. CNN is one of the most popular AI models in the field of image recognition right now. Compared with the traditional artificial neural network method, the structure of CNN is simpler and can save more computing resources. At present, CNN models can identify people with masks very well^16^.

We annotated 1000 images from the collected images as the training dataset for training our model. At first, the MATLAB 2020a has been used to annotate the training dataset. We annotated each head in the image and generated a map which has the same size as the original image. In the annotated map, the pixel value at the annotation is one and others pixel value is zero. Then, we use a Gaussian kernel with a kernel size is 15 and σ is 4 to perform Gaussian filtering on the annotated map to obtain the density map as the ground truth of our dataset. The total pixel value of the density map is the total number of people in the image.

We build a Multi-fusion convolutional neural network (MFCNN) based on the ResNet^17^ and U-Net^18^ to predict the number of people in the images. Our MFCNN uses the encoder–decoder structure. In the encoder, our MFCNN has an input block and four downsampling blocks. The input block condenses the image to a quarter of its original size. This can reduce the amount of memory consumed by the model at runtime. The feature image then passes through four consecutive downsampling blocks. In order from shallow to deep, these blocks gradually include more base blocks to enhance the feature extraction ability. The base block is built based on the bottleneck layer of ResNet. In decoder, the feature map in the depth would be upsampled and fused with the feature map with larger size. Finally, fine-grained regressor refines the feature map and generates a density map to predict the number of people.

In the fine-grained compressor, the input feature map is cut into strips from the width and height dimensions. Each strip is sent to a base block for fine-grained local feature extraction. This step can increase the ability of extracting the correlation between features to obtain a more complete feature image. Then, these strips are spliced into a feature image through the width and height dimensions. Feature maps with fine-grained feature enhancement in the two dimensions are fused as the final feature map. The regression layer composed of a global average pooling layer and a convolutional layer predicts the density map through the final feature map. The predicted density map is enlarged to the size of the input image by a dilated convolutional layer. We use global average pooling layer and 1 × 1 convolutional layer to replace the fully connection layer. This enables our model to adapt to various input images of different sizes.

**Figure 1:**
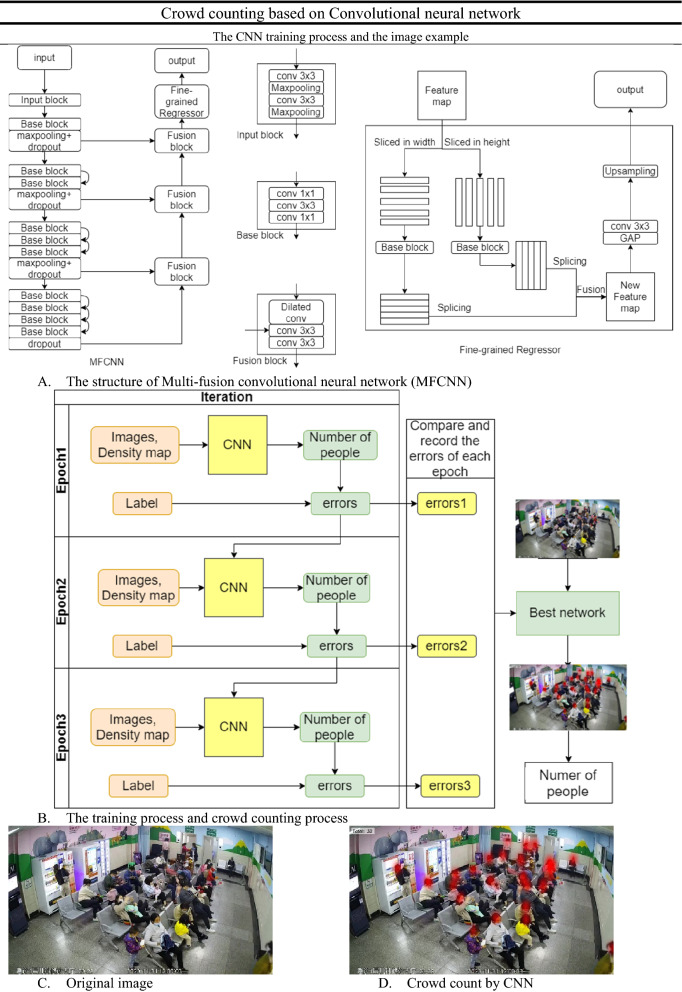
Crowd counting based on convolutional neural network.

The training process is shown in Fig. 1. In each epoch, the system records training errors and corrects the network in the next epoch. After training, the system compares the errors from each epoch and picks the network with the best results. Finally, the best network for crowd counting can help us get the number of people from images. Our experiments are based on a Python environment. Batch size is set to 8, Adam is the optimizer, and 50 epochs are learned at a fixed learning rate of 1e−5.

### Statistics

#### Statistics on CNN model

We use two indexes to evaluate the prediction results to predict the number of people. The mean counting error (MCE), which measures instance counting accuracy for R images:

1 $$MCE=\frac{1}{R}\sum_{i=1}^{R}\left|{C}_{gt}^{i}-{C}_{pred}^{i}\right|,$$

where ${C}_{gt}^{i}$ represents the ground truth number of people in the *i*-th picture, and ${C}_{pred}^{i}$ is the number of people predicted by the generator. MCE can numerically represent the average number of false identifications per image. A smaller value of MCE means a smaller amount of counting error per image on average.

The root mean squared error (RMSE) can reflect the deviation value of the prediction error of each instance:

2 $$RMSE=\sqrt{\frac{1}{R}\sum_{i=1}^{R}{\left({C}_{gt}^{i}-{C}_{pred}^{i}\right)}^{2},}$$

where *R* is the number of responses, ${C}_{gt}^{i}$ represents the ground truth number of people in the *i*-th picture, and ${C}_{pred}^{i}$ is the number of people predicted by the generator. RMSE is a commonly used model performance evaluation metric in the field of object counting. A smaller value of RMSE means better accuracy of the model prediction.

#### Statistics on numbers of people

Based on the obtained crowd data, from the two perspectives of different areas at the same time and the same area at different times, the crowd information, which is collected from the images, is sorted by date. First, we identified the number of people in every image by CNN for each sampling time point. The number of people from all pictures at this sampling time point is then averaged to get the number of people at this sampling time point in one area. The average number of individuals at each site is finally counted into a table in chronological order.

#### Statistics on time spent

The emergency department visit records are processed to calculate the length of visit time after diagnosis (*T*1) and the length of diagnosis time (*T*2). *T*1 is the time of the earliest drug delivery time or the earliest payment time minus the time of pickup. *T*2 is the earliest time to diagnosis minus the time of pickup. After that, we extracted the influencing factors that might be associated with *T*1 and *T*2 from the records of emergency department visits: gender, age, number of diseases, and kind of diseases.

#### Unit congestion rate

According to Hall’s Personal Space Theory of Social Psychology, personal distance is usually defined as 4 feet, or 1.2 m^19^. Therefore, we can estimate the theoretical number of people awaiting treatment in an area based on the area of this location and the personal distance, which makes the waiting crowd feel psychological discomfort, as shown in Eq. (3). Then take the ratio of the actual number of people to the theoretical capacity as the unit congestion rate in the area, as shown in Eq. (4).

3 $${N}_{theoretical}= \frac{area}{{\pi d}_{personal}^{2}},$$

4 $${R}_{c}=\frac{{N}_{actual}}{{N}_{theoretical}}.$$

Based on the unit congestion rate, it fits the curve graph of the congestion rate change over time in each position within a day; draws the fluctuation diagram of the unit congestion rate change along the medical process at each time point; draws the fluctuation diagram of the unit congestion rate change on each medical process at the same time on different days of a week.

#### Influence factor

In addition, we use t-test, ANOVAs test and Kruskal Wallis test to investigate the correlation of changes in visit length or diagnosis time with several influencing factors. Explored whether these influencing factors could contribute to hospital congestion by affecting the length of a visit or diagnosis time.

All analyses are performed using the social science statistics package (SPSS) version 25 (SPSS, Chicago, USA). First, the descriptive statistics (frequency, percentage, average and standard deviation [SD]) are calculated. We use T-test to compare the 2 groups. And ANOVAs test is performed for multi-group comparison to evaluate the difference of continuous variables, when the data is the normal distribution and conforms to the assumption of homogeneity of variance. In addition, the non-parameter method is adopted. When the test of variance shows heterogeneity of variance, we use the Kruskal–Wallis H test to find the relationship between groups. The probability value of P < 0.05 means statistically significant.

### Ethical statement

All methods are performed in accordance with the relevant guidelines and regulations by including a statement in the “Methods” section. The acquisition of surveillance video and building data is approved by the hospital and approved by the ethics committee. The acquisition of visit records is approved by the participating hospitals and all patients. Informed consent is obtained by telephone from the participants. For patients aged < 18 years, consent is obtained from a legal guardian. The data are all processed for desensitization, and low-resolution images are used for video images. The research has approval from the ethics committee of the Shanghai Jiaotong University School of medicine.

## Result

The hospital record has 4717 records, of which 1042 records can not count the length of diagnosis time or the length of visit time after diagnosis, and 3675 records are useful, accounting for 77.9% of the total.

### Validation of multi fusion convolutional neural network

We use 1000 annotated images to train our multi fusion convolutional neural network. 600 images, of which 600 are used for model training and 400 for model testing. The division of test and training sets is random. Mall dataset contains 2000 images. We randomly choose 1600 images for model training and 400 for model testing.

We compared our MFCNN with some classical CNN model on our dataset and Mall dataset, which is a public dataset and has a similar scenario to our dataset. In Table 1, the experimental results show that our MFCNN has the lowest mean counting error (MCE) and root mean squared error (RMSE), which means our MFCNN has the better counting accuracy than the classical CNN model.

**Table 1:** The comparison of MFCNN and other models on crowd counting.

Datasets	Mall dataset		Our dataset	
Models	MCE	RMSE	MCE	RMSE
ResNet 50^17^	2	2.6	1.91	2.43
AlexNet^20^	1.95	2.46	1.83	2.4
VGG 16^21^	1.94	2.47	1.88	2.42
MFCN (ours)	1.6	2.1	1.4	1.87

### Distribution of waiting people in surveillance video

**Figure 2:**
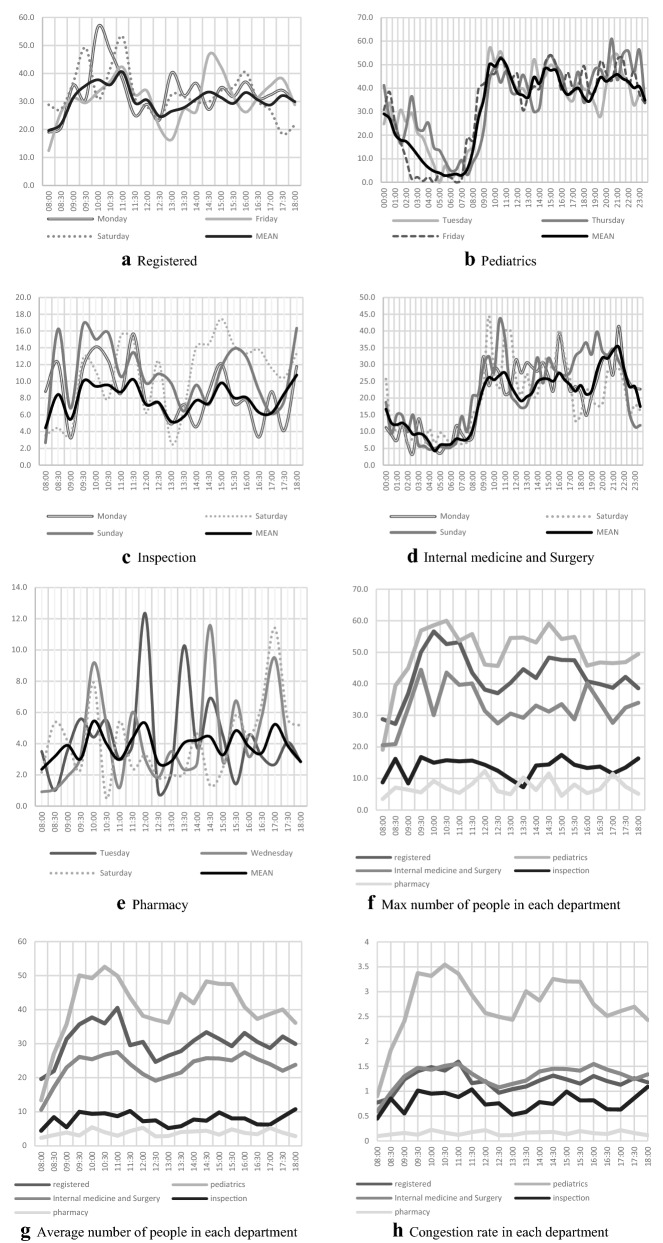
Trend of people in each sampling area of ER in 1 week.

Using a convolution neural network, we calculate the number of people waiting in the waiting area of each department, and the results are recorded in Table 2 (Details shown in Supplementary file) and Fig. 2. The waiting numbers are similar on each day of the week. The cumulative number of people waiting for treatment in pediatric waiting areas is the highest, totaling 10,436, accounting for 45% of the total number of people waiting for treatment approximately. The pharmacy has a minimum number of people waiting for treatment of 566 people, approximately 2%.

**Table 2:** Distribution of people in each sampling area in 1 week.

	Registered	Pediatrics	Internal medicine and Surgery	Inspection	Pharmacy	Sum
Monday	693	1485	963	183	75	3399
Tuesday	599	1584	850	136	91	3260
Wednesday	637	1387	902	169	89	3184
Thursday	582	1639	869	103	78	3271
Friday	650	1541	944	114	68	3317
Saturday	682	1335	947	225	87	3276
Sunday	647	1465	1037	229	78	3456
Sum	4490	10,436	6512	1159	566	23,163

For Registration, the maximum peak of waiting number in the morning occurred on Monday and it in the afternoon occurs on Friday; There is a small peak almost every morning at 11:00; The maximum peak of waiting people occurs later on weekends than it on weekdays.

For Pediatrics, the number of people waiting for treatment increase rapidly from 8:30 am to 9:30 and maintained a high level throughout the day, with a smaller decrease in waiting numbers at midday and afternoon rest times; Usually, the number of people waiting for treatment on weekend nights is smaller than that on weekday nights. For the waiting areas of Internal medicine and Surgery department, the number of people waiting for treatment is large and fluctuating.

In inspection, the first peak of waiting people on each day comes earlier than it in other departments, usually around 8:30; And the waiting number has a rapid upstroke before the emergency department knocking off at 18:00. There is not a large gap in the total reception number per day at the pharmacy, but waiting numbers in various periods of the day fluctuated greatly. Although the average waiting number in the registered area is smaller than that of Pediatrics, its maximum waiting number is close to that of Pediatrics. Crowdedness is noted in all areas except testing departments and pharmacies, where crowdedness is greatest in Pediatrics waiting areas.

### Factors affecting the length of diagnosis time and visit time after diagnosis

**Figure 3:**
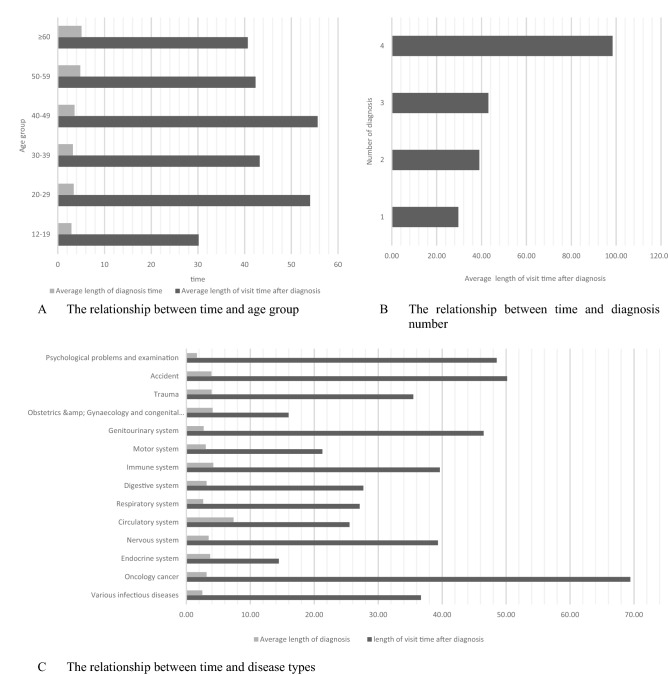
Planning information for emergency departments.

In Table 3 and Fig. 3, based on the available data, we have listed 4 factors that may affect the length of visit time after diagnosis and diagnosis time: age, gender, number of diagnoses, disease types. Of the patients, 66% are children under ten years of age. We make separate comparisons between groups of children. The period under 1-year-old is called infancy, the period from 1 to 3 years old is called early childhood, the period from 3 to 6 years old is called pre-school, and the period from 6 to 12 years old is called school age. Only one disease has been diagnosed in most patients. The most common comes from respiratory problems, accounting for 54.3%.

**Table 3:** Basic demographic characteristics and influence factors.

Items	Groups		Number	Percentage (%)	P-value	
					Length of visit time after diagnosis	Length of diagnosis time
Sex	Male		1889	52.03	0.528	0.335
	Female		1741	47.97		
Ages	Children	< 1	288	7.84	0.77	0.17
		1–2	721	19.62		
		3–5	1007	27.40		
		6–12	457	12.44		
	Teenagers and adults	12–19	178	4.84	< 0.001	< 0.001
		20–29	195	5.31		
		30–39	221	6.01		
		40–49	158	4.30		
		50–59	190	5.17		
		≥ 60	260	7.07		
Number of diagnoses	1		2853	78.8	0.028	0.2
	2		727	20.1		
	3		37	1.0		
	4		3	0.1		
Disease types	Various infectious diseases		151	4.0	< 0.001	< 0.001
	Oncology cancer		2	0.1		
	Endocrine system		7	0.2		
	Nervous system		29	0.8		
	Circulatory system		32	0.9		
	Respiratory system		2036	54.3		
	Digestive system		396	10.6		
	Immune system		525	14.0		
	Motor system		21	0.6		
	Genitourinary system		64	1.7		
	Obstetrics and congenital disorders		10	0.3		
	Trauma		200	5.3		
	Accident		78	2.1		
	Psychological problems and examination		197	5.3

### Architecture distribution

In Table 4, we obtain the architectural data for this emergency in the diagnostic area through building drawings and field surveys and we calculate the Optimal number of people waiting for treatment for the area based on Personal Space Theory^19^. Among them, the registered lobby has the largest building area. And Monday and Friday have the highest number of doctors arranged.

**Table 4:** Planning information for emergency departments.

	Registered	Pediatrics	Internal medicine and Surgery	Inspection	Pharmacy
Area (m^2^)	115.02	67.14	66.78	44.73	110.24
Number of seats	12	30	16	11	0
Optimal number of people waiting for treatment	25.42	14.84	17.76	9.88	24.36
Average number of people waiting for treatment	30.54	40.75	23.19	7.84	3.82
Average congestion rate	1.2	2.75	1.3	0.79	0.16

## Discussion

It is important to optimize the allocation of health resources. Current tertiary hospitals in China also commonly suffer from the problems of ‘three long and one short’ (long registration time, long waiting time, long time to pick up drugs, and short visiting time). Through the study of hospital congestion, it is helpful for managers to understand the flow of patients in the hospital. Thus, it can improve the configuration of resources and the visiting service of hospitals.

The survey shows that the burden in Pediatrics is substantial. Pediatrics had the largest waiting list and the greatest patient density. The waiting number of Pediatrics rose rapidly during the morning hours from 8 to 9 am and is maintained around 40 thereafter. In terms of the congestion rate, the average congestion rate of Pediatrics is 2.75^19^. In fact, the pediatric environment is also chaotic, and the affected children often cry loudly. This environment would aggravate the anxiety of patients and their families and thus reduce the patient’s visiting experience and satisfaction^22^. Additionally, the emphasis placed on the next generation in Chinese traditional culture has been easily magnified by the parents’ anxiety from the malaise of child visits, which further worsens the diagnosis and treatment experience. Poor experience of patient may lead to decreased medical effectiveness and even causes Doctor–patient Conflict^23,24^. Increasing the number of physicians to reduce the peak waiting number might be an effective way to improve their experience. However, at present, pediatrics is a subject that doctors are reluctant to choose. The lack of availability of pediatricians has become increasingly significant. Government and hospitals should improve the treatment of pediatrics to increase the number of pediatricians. Additionally, providing possible length of waiting time can help to enhance the visitor’s experience^25,26^. At the same time, expanding the area of the waiting region to reduce the density of the waiting people may improve the patient's experience. Moreover, improving the environment of pediatric, including recreational imaging, wallpaper color, children’s activity areas and animation to divert the child’s attention, might reduce the crying of the child and relieve the anxiety of the child’s family^22^.

The medical examination is an important link in the modern medical process and inspection reports are the important basis for physicians to initiate diagnosis and treatment. It should be noticed that the distribution of waiting people in the Inspection shows a dumbbell type distribution: high at both heads and low in the middle. The first peak of waiting number in the Inspection comes earlier than it in other departments, which is usually at 8:30, while the others are at 9:30. In addition, the number of waiting people at the Inspection is rise before off-hours, which may indicate that patients are eager to complete the medical test before off hours to avoid dragging the test to the next day. Previous studies have shown that sampling examinations are required for the majority of patients, and the waiting time for sampling examinations is long^27^. In Table 3, concurrence of multiple diseases does not result in a longer diagnostic time for physicians, but significantly increases the length of time used after diagnosis. Because multiple diseases are possible, the patient is referred for multiple medical tests to further define the type and circumstances of the disease. So doctors need to rely on laboratory examinations to detect the patient’s condition, and complex diseases spend significantly more time on inspection to be determined like Oncology cancer^28^. The speed of examination constrains the speed of the whole visit process. The main reason for the patients waiting in the queue at the Inspection department is waiting for samples collection. Too long waiting time could affect the fluency of all visit progress and cause patient dissatisfaction^22,29^. Faced with the above problems, on the one hand, hospitals may implement a shift by improving the scheduling mechanism, changing the scheduling time of the laboratory department, increasing the number of workers in the morning and off-hours, or by adding a shift to reduce the waiting time of patients in the queue and avoid the centralized outbreak of inspection needs. On the other hand, the hospital could improve testing devices^30,31^. The use of more advanced equipment can reduce the time required for a single inspection. In addition, by conducting a health economics analysis of devices’ effectiveness, hospitals can comprehensively judge how much unit time inspection efficiency and hospital operation efficiency have been improved by the introduction of high-efficiency equipment^32^.

Meanwhile, pharmacies with very low crowding rates also deserve attention. As can be seen in Table 4, the independent emergency pharmacy is less frequently used but occupies a significant amount of space. Other studies have shown that pharmacies are indeed a vulnerable, wasteful and inefficient link in hospitals^33^. The emergency pharmacy could be combined with the outpatient department pharmacy. In this way, the hospital carries on the unified planning to the pharmacy, reduces resources waste, and improves the working efficiency^34^. On the other hand, hospitals can cooperate with outside drugstores. So that some patients can choose to buy drugs in drugstores. This is helpful in shortening the total time of patients in the hospital and reduce hospital expenses (Supplementary Information).

In Fig. 3, there is a clear positive correlation trend between patient age and length of diagnosis time. This may be related to disease complexity in elder patients^35^. Expressive skills and memory are relatively poor in the elderly compared to the young^36,37^. Physicians may spend more time communicating to determine the type of condition and disease development when faced with an elder patient. For the elder patients, we can strengthen the role of the family doctors, let them join the consultation and make use of well-established case records so that the patient's appeal can be more clearly expressed^38,39^. Nevertheless, younger patients generally spend more time after diagnosis than elder patients. Because most elder patients are Geriatric disorders or Chronic diseases patients, and they regularly visit the hospital for diagnosis^40^. Therefore, these patients are generally more familiar with the department setting and visit process, while young patients may not be familiar enough. In response to the issue of inexperienced access for young people, we suggest that an introduction to the visit process can be provided on the appointment software to help young people understand the visiting process in the hospital and the location of each department. On the other hand, the hospital can set some striking signals or electronic navigation, which facilitates patients to find destinations quickly^41^.

In addition, respiratory diseases occupy more than 50% of the patients in the emergency department and most of them are influenza patients. This leads to congestion in the emergency department and a waste of medical resources. Most influenza patients could choose to go to community hospitals rather than tertiary hospitals. From other studies, we found that this is a common phenomenon that patients are more inclined to seek treatment in tertiary hospitals rather than community hospitals^15^. We believe that the local government also needs to strengthen the construction of community hospitals, strengthen the training of family doctors, and improve the hierarchical diagnosis and treatment system^39,42^. Through community hospitals, family doctors and other means, to relieve the crowdedness of central hospitals and to reduce the time-consuming of patients^43^.

## Conclusion

This study presents a summary analysis of congestion at a tertiary hospital through a survey based on crowd counting via CNN. We propose a process of medical treatment based on hospital visit flow to divide the regions of the hospital. Meanwhile, we propose the unit congestion rate, which is used to calculate the crowdedness of a location. To count the number of waiting people, we propose the multi fusion convolutional neural network. Through the statistical analysis of the monitoring data by artificial intelligence, we validate the congestion existing at this hospital. Based on this, we propose improvements in the allocation of medical resources to the hospital. In addition, this study finds associations of age, disease category with the length of diagnosis time, and length of time after diagnosis. And we also find a relation between the number of diagnoses and length of time after diagnosis. These empirical data also help us to make improved opinions on the allocation of medical resources in this hospital and local medical policies for the local government.

However, the sample size is not large enough due to the short period of data acquisition. Some possible periodic features may fail to be detected. And our failure to access the appointment registration to face-to-face visits, so we are not able to conduct a more complete study of the whole visit flow. In the future, we will conduct more research to better play the role of AI in medical management.

## Supplementary Information

Supplementary Information.

## Data Availability

The datasets use and/or analyse during the current study are available from the corresponding author on reasonable request.

## References

[1] Pan X, Zhu S (2020). Development of health resources allocation in Sichuan province. Sustain. Dev..

[2] Marć M, Bartosiewicz A, Burzyńska J, Chmiel Z, Januszewicz P (2019). A nursing shortage—A prospect of global and local policies. Int. Nurs. Rev..

[3] Otani S, Majbauddin A, Kurozawa Y, Shinoda M (2018). Lack of medical resources and public health vulnerability in Mongolia's winter disasters. Rural Remote Health.

[4] Zhang X, Tai D, Pforsich H, Lin VW (2018). United States registered nurse workforce report card and shortage forecast: A revisit. Am. J. Med. Qual.

[5] Sun Z, Wang S, Barnes SR (2016). Understanding congestion in China's medical market: An incentive structure perspective. Health Policy Plan.

[6] Chou S-C, Baker O, Schuur JD (2020). Changes in emergency department care intensity from 2007–2016: Analysis of the national hospital ambulatory medical care survey. West. J. Emerg. Med..

[7] Salmon S, McLaws ML (2015). Environmental challenges of identifying a patient zone and the healthcare zone in a crowded Vietnamese hospital. J. Hosp. Infect..

[8] Chang AM (2018). Hospital strategies for reducing emergency department crowding: A mixed-methods study. Ann. Emerg. Med..

[9] Shultz J (2020). Simulation-based mock-up evaluation of a universal operating room. HERD.

[10] Haskins, G., Kruger, U. & Yan, P. Deep learning in medical image registration: A survey. Preprint at https://ui.adsabs.harvard.edu/abs/2019arXiv190302026H; 10.1007/s00138-020-01060-x.

[11] Chinzei K (2018). Regulatory science on AI-based medical devices and systems. Adv. Biomed. Eng..

[12] Alnabulsi H, Drury J (2014). Social identification moderates the effect of crowd density on safety at the Hajj. Proc. Natl. Acad. Sci..

[13] Allam Z, Jones DS (2020). On the coronavirus (COVID-19) outbreak and the smart city network: Universal data sharing standards coupled with artificial intelligence (AI) to benefit urban health monitoring and management. Healthcare.

[14] Skobelev P, Demazeau Y, An B, Bajo J, Fernández-Caballero A (2018). Towards autonomous AI systems for resource management: Applications in industry and lessons learned. Advances in Practical Applications of Agents, Multi-Agent Systems, and Complexity: The PAAMS Collection.

[15] Oh HC, Chow WL, Gao Y, Tiah L, Goh SH, Mohan T (2020). Factors associated with inappropriate attendances at the emergency department of a tertiary hospital in Singapore. Singap. Med. J..

[16] Singh A, Jindal V, Sandhu R, Chang V (2022). A scalable framework for smart COVID surveillance in the workplace using deep neural networks and cloud computing. Expert Syst..

[17] He, K., Zhang, X., Ren, S. & Sun, J. Deep residual learning for image recognition. In *2016 IEEE Conference on Computer Vision and Pattern Recognition (CVPR)*, 770–778 (2016).

[18] Ronneberger O, Fischer P, Brox T (2015). U-Net: Convolutional Networks for Biomedical Image Segmentation.

[19] Hall ET (1966). The Hidden Dimension.

[20] Krizhevsky, A., Sutskever, I. & Hinton, G. E. ImageNet classification with deep convolutional neural networks. In *Presented at the Proc. 25th International Conference on Neural Information Processing Systems, Lake Tahoe, Nevada*. 10.5555/2999134.2999257 (2012).

[21] Zhang X, Zou J, He K, Sun J (2016). Accelerating very deep convolutional networks for classification and detection. IEEE Trans. Pattern Anal. Mach. Intell..

[22] Xie Z, Or C (2017). Associations between waiting times, service times, and patient satisfaction in an endocrinology outpatient department: A time study and questionnaire survey. Inquiry.

[23] Roney LN, Acri MC (2018). The cost of caring: An exploration of compassion fatigue, compassion satisfaction, and job satisfaction in pediatric nurses. J. Pediatr. Nurs..

[24] Qiao T, Fan Y, Geater AF, Chongsuvivatwong V, McNeil EB (2019). Factors associated with the doctor-patient relationship: Doctor and patient perspectives in hospital outpatient clinics of Inner Mongolia Autonomous Region, China. Patient Prefer. Adher..

[25] Ma WM, Zhang H, Wang NL (2019). Improving outpatient satisfaction by extending expected waiting time. BMC Health Serv. Res..

[26] Rönnerstrand B, Oskarson M (2020). Standing in line when queues are on the decline: Services Satisfaction following the Swedish health care waiting time guarantee. Policy Stud. J..

[27] Ahmad J, Iqbal J, Ahmad I, Khan ZA, Tiwana MI, Khan K (2020). A simulation based study for managing hospital resources by reducing patient waiting time. IEEE Access.

[28] Jacobsen MM (2017). Timeliness of access to lung cancer diagnosis and treatment: A scoping literature review. Lung Cancer.

[29] Sun J (2017). Reducing waiting time and raising outpatient satisfaction in a Chinese public tertiary general hospital-an interrupted time series study. BMC Public Health.

[30] Farzaneh T (2019). Crucial role for pathology residents in laboratory self-inspection, a single Institute's experience. Pract. Lab. Med..

[31] Guo Y, Chen Y, Lane DA, Liu L, Wang Y, Lip GYH (2017). Mobile health technology for atrial fibrillation management integrating decision support, education, and patient involvement: mAF app trial. Am. J. Med..

[32] Kisser A, Tüchler H, Erdös J, Wild C (2016). Factors influencing coverage decisions on medical devices: A retrospective analysis of 78 medical device appraisals for the Austrian hospital benefit catalogue 2008–2015. Health Policy.

[33] Malet-Larrea A (2019). Defining and characterising age-friendly community pharmacies: A qualitative study. Int. J. Pharm. Pract..

[34] González-Álvaro I (2015). Spanish Rheumatology Society and Hospital Pharmacy Society Consensus on recommendations for biologics optimization in patients with rheumatoid arthritis, ankylosing spondylitis and psoriatic arthritis. Rheumatology.

[35] Golovanova ED (2020). Prevalence and features of geriatric syndromes treatment in old patients (clinical and epidemiological study). Adv. Gerontol..

[36] Abichou K, La Corte V, Nicolas S, Piolino P (2020). Les faux souvenirs dans le vieillissement normal: Données empiriques du paradigme DRM et perspectives théoriques (False memory in normal aging: Empirical data from the DRM paradigm and theoretical perspectives). Geriatr. Psychol. Neuropsychiatr. Vieil.

[37] Mormer E, Bubb KJ, Alrawashdeh M, Cipkala-Gaffin JA (2020). Hearing loss and communication among hospitalized older adults: Prevalence and recognition. J. Gerontol. Nurs..

[38] Homann K, Bertsche T, Schiek S (2021). Pharmacy technicians' perception about symptoms and concerns of older patients visiting pharmacies: A cross-sectional study. J. Multidiscip. Healthc..

[39] Schafheutle EI, Jee SD, Willis SC (2017). Fitness for purpose of pharmacy technician education and training: The case of Great Britain. Res. Soc. Adm. Pharm..

[40] Katsoulis J, Huber S, Mericske-Stern R (2009). Gerodontologischer Konsiliardienst bei stationären Geriatriepatienten: Allgemeinmedizinischer Zustand (I) (Gerodontology consultation in geriatric facilities: General health status (I)). Schweiz Monatsschr Zahnmed.

[41] Ženka J, Macháček J, Krtička L, Michna P, Kořízek P (2021). Acceptance of a smartphone navigation application by hospital patients and visitors: The role of gender, age, and education. Hung. Geogr. Bull..

[42] Zhu D, Shi X, Nicholas S, Bai Q, He P (2019). Impact of China's healthcare price reforms on traditional Chinese medicine public hospitals in Beijing: An interrupted time-series study. BMJ Open.

[43] O'Mahony C (2020). A cost comparison study to review community versus acute hospital models of nursing care delivered to oncology patients. Eur. J. Oncol. Nurs..

